# Evaluation of an Optical Defocus Treatment for Myopia Progression Among Schoolchildren During the COVID-19 Pandemic

**DOI:** 10.1001/jamanetworkopen.2021.43781

**Published:** 2022-01-14

**Authors:** Kai Yip Choi, Rachel Ka Man Chun, Wing Chun Tang, Chi Ho To, Carly Siu-yin Lam, Henry Ho-lung Chan

**Affiliations:** 1 Centre for Myopia Research, School of Optometry, The Hong Kong Polytechnic University, Hong Kong; 2 Research Centre for SHARP Vision, The Hong Kong Polytechnic University, Hong Kong; 3 Centre for Eye and Vision Research, Hong Kong; 4 University Research Facilities in Behavioral and Systems Neurosciences, The Hong Kong Polytechnic University, Hong Kong

## Abstract

**Importance:**

Myopia progression has been found to be worsening during the COVID-19 pandemic. It is important to control the rapid myopia progression in this period.

**Objective:**

To analyze the association of COVID-19–related lockdown measures with myopia progression in schoolchildren and to compare the performance of defocus incorporated multiple segments (DIMS) lens with that of single vision lens (SVL) treatment in reducing myopia progression.

**Design, Setting, and Participants:**

This cohort study involved an exploratory, prespecified, comparison of 2 independent longitudinal studies performed at the same institute beginning in 2019. Data from Hong Kong schoolchildren (aged 7-13 years) were gathered and analyzed. Data analysis was performed from June to July 2021.

**Exposure:**

Schoolchildren in study 1 wore a DIMS lens for 18 months, and those in study 2 wore a SVL for 24 months.

**Main Outcomes and Measures:**

Cycloplegic spherical equivalent refraction and axial length were measured. Studies 1 and 2 started before the start of lockdown measures and continued throughout the lockdown. In both studies, periods of fewer and more COVID-19–related lockdown measures were identified. Because COVID-19 lockdown caused deviations from the visit schedule, myopia progression was normalized to 12-month change, which were compared between DIMS and SVL groups, also during the periods with less and more lockdown time.

**Results:**

There were 115 participants (58 girls [50.4%]; mean [SD] age, 10.3 [1.5] years) in the DIMS group; their mean (SD) baseline refraction was −4.02 (1.46) D. There were 56 participants (29 girls [51.8%]; mean [SD] age, 10.8 [1.5] years) in the SVL group; their mean (SD) baseline refraction was −2.99 (1.06) D. After controlling for the covariates, DIMS treatment was significantly associated with 34% less axial elongation (0.19 mm [95% CI, 0.16 to 0.22 mm] vs 0.30 mm [95% CI, 0.25 to 0.35 mm]; *P* < .001) and 46% less myopic progression after 12 months (−0.31 D [95% CI, −0.39 to −0.23 D] vs −0.57 D [95% CI, −0.69 to −0.45 D]; *P* = .001) compared with SVL treatment. In both the DIMS and SVL groups, more lockdown time was associated with significantly more spherical equivalent refraction (−0.54 D [95% CI, −0.64 to 0.44 D] vs −0.34 D [95% CI, −0.44 to −0.25 D]; *P* = .01) and axial length (0.29 mm [95% CI, 0.25 to 0.32 mm] vs 0.20 mm [95% CI, 0.16 to 0.24 mm]; *P* = .001) compared with less lockdown time. No significant interaction between treatment type and lockdown time was observed.

**Conclusions and Relevance:**

In this exploratory analysis, myopia progressed more rapidly in schoolchildren during the period when there were more COVID-19–related lockdown measures. However, optical treatment with DIMS was significantly associated with slower myopia progression compared with SVL treatment during the lockdown period.

## Key Points

**Question:**

Is an optical defocus treatment associated with slowed myopia progression among schoolchildren experiencing lockdown related to the COVID-19 pandemic?

**Findings:**

In this exploratory analysis of 2 cohort studies including 171 schoolchildren during COVID-19 lockdown, treatment using a defocus incorporated multiple segments lens was associated with 46% less myopia progression and 34% less axial elongation compared with regular single vision lens treatment.

**Meaning:**

These findings suggest that an optical defocus treatment may be associated with slower myopia progression, which has been exaggerated during the COVID-19 pandemic, among schoolchildren.

## Teaser

This cohort study analyzes the association of COVID-19–related lockdown measures with myopia progression in schoolchildren using data from 2 independent longitudinal studies and compares the performance of defocus incorporated multiple segments lens with single vision lens in reducing myopia progression.

## Introduction

The COVID-19 pandemic, which is caused by the novel coronavirus SARS-CoV-2, began in December 2019 and has infected more than 100 million individuals and caused millions of deaths globally.^1^ The World Health Organization declared COVID-19 a global pandemic a few months after the initial outbreak. To restrict and prevent the spread of the pandemic, public health measures, including mandatory mask wearing, social distancing, work-from-home policies, implementation of online school classes, and travel restrictions, have been imposed by governments all over the world.

During the pandemic, children spent more time at home because of suspension of face-to-face teaching at school in favor of online learning.^2^ It is reasonable to expect that these measures led to an increase in near work activities for children (digital or otherwise) and substantially less time spent outdoors.^3,4,5^ Generally, time spent on near work is positively correlated with myopia progression, whereas time spent outdoors is negatively correlated with myopia.^6,7,8,9,10^ Overall, during the periods of lockdown, children spent most of their time at home—that is, their refractive development was overwhelmingly determined by their domestic living environment.^10,11,12^ In view of these considerations, it is reasonable to expect increased myopia progression in schoolchildren during the pandemic due to the lockdown-imposed behavioral and living style changes.^13^ Recent longitudinal cohort studies^14,15^ also demonstrated an increased myopia progression in children during the lockdown.

The prevalence of myopia is particularly high in East Asian areas, such as China, Taiwan, Singapore, and Hong Kong.^16^ The prevalence is anticipated to further increase worldwide. Approximately 50% of the world’s population could have myopia by the year 2050.^17^ Individuals with myopia, especially those with high myopia, are more prone to sight-threatening diseases, such as glaucoma and retinal degenerations.^18^ To tackle the rapid progression and prevent the occurrence of myopia, different strategies have been explored for myopia control in children with risk factors for myopia progression.^19,20,21,22^ One of those strategies makes use of myopic optical defocus, which is imposed by a positive powered lens that casts a focal plane in front of the retina. Myopia progression has been effectively controlled by myopic defocus in animal models and in humans.^21,23,24,25,26,27^ Recently, a novel spectacle lens (defocused incorporated multiple segments [DIMS]) that incorporates 3.50 D myopic defocus with multiple lenslets at peripheral field was designed. A double masked and randomized clinical trial^27^ has demonstrated significant protective effect from myopia progression in schoolchildren using the DIMS spectacle lens. However, whether the DIMS lens is still effective in controlling myopia under the high-risk (eg, indoors-rich and outdoors lacking) environment created by the pandemic remains unclear.

Although a previous cross-sectional study^28^ revealed an increase in myopia prevalence and incidence during the COVID-19 pandemic compared with historical numbers and some recent studies^14,15^ longitudinally following up total cohorts of children, the current study leveraged data from longitudinal studies that were prospectively conducted during the pandemic in school-aged children with myopia requiring optical correction. Using exploratory, prespecified analyses, the association of lockdown measures on myopia progression was evaluated, in addition to the performance of DIMS spectacle lenses for control of myopia progression during the COVID-19–induced lockdown period.

## Methods

Data from 2 studies were used for the current cohort study. In study 1, DIMS lens treatment was prescribed to children in need who underwent regular eye examinations annually for 2 years. The study was funded as a community project. Study 2 was a randomized clinical trial (ClinicalTrials.gov registration number NCT03538002) from which only the control group wearing single vision lens (SVL) was included in the current analysis. Both studies recruited schoolchildren aged 7 to 13 years and were conducted in Hong Kong and largely in parallel, with study participants undergoing scheduled eye examinations at the Optometry Research Clinic, The Hong Kong Polytechnic University. The current study follows the principles of the Declaration of Helsinki^29^ and has been approved by the Human Subjects Ethics Subcommittee of the Hong Kong Polytechnic University. The DIMS lens community project was a charity-based intervention that did not require approval from the institutional review board. Nevertheless, written consent and verbal assent were obtained from the parents and the participants, respectively, who consented to any research use of the eye examination results. This study follows the Strengthening the Reporting of Observational Studies in Epidemiology (STROBE) reporting guideline.

The Hong Kong government suspended all face-to-face school classes and activities from February 2020, because of the worsening of the COVID-19 pandemic, until May 2021.^30^ To evaluate the association of the lockdown with outcomes in both studies and to avoid bias resulting from comparing different time periods, only visits from June 2019 onward were included in the analyses. As a result, in study 2 (SVL), all baseline visits and some of the first postbaseline visits were omitted for the current analyses, and subsequent visits were used for rebaselining, whereas all visits performed in study 1 (DIMS) were available for analysis. Because of the COVID-19 lockdown in Hong Kong, in both studies, deviations for timing of the study visits were quite common. To account for the nonuniform study intervals, change from baseline in axial length (AL) and spherical equivalent refraction (SER) were proportionally adjusted to a 12-month change. The first visit from June 2019 onward was treated as baseline (rebaselining), and the subsequent follow-up visits were reviewed for refractive changes. Visits from 10 months and onward up to 19 months from baseline were used, and the normalization was calculated as follows: change in SER or AL × (12 ÷ months between baseline and follow-up).

**Figure 1.**
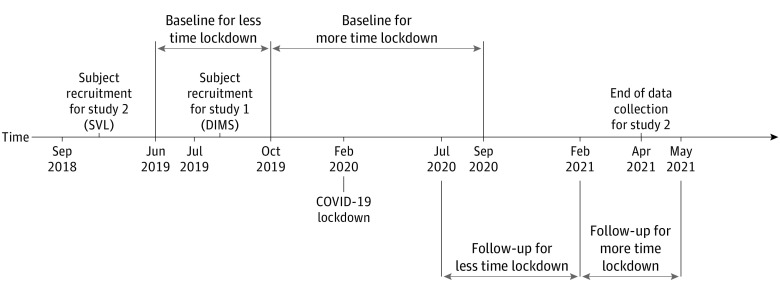
Timeline for Baseline and Follow-up Schedules DIMS indicates defocus incorporated multiple segments; SVL, single vision lens.

To evaluate the association of the lockdown with study outcomes, the lockdown period was further categorized according to the time point of the baseline and follow-up visits. Because participants with baseline visits early after June 2019 were likely to have spent less time in lockdown up to the follow-up visit, the lockdown severity (or time spent in lockdown) was greater for participants with late baseline visits. To account for this phenomenon, the study populations were divided according to lockdown severity (ie, less time vs more time) by median-splitting the percentage of time lockdown in between baseline and follow-up visits. The timeline for follow-up schedules is shown in Figure 1, and the number of participants, follow-up schedule, and percentage of time lockdown are summarized in Table 1.

**Table 1.** Baseline Characteristics of Participants in DIMS and SVL Studies by Lockdown Severity

Treatment type and lockdown time	Sex, No. of participants		Age, mean (SD), y^a^	Time from baseline to follow-up, median (IQR), mo	Time in lockdown, mean (SD), %^b^	Mean (95% CI)	
	Female	Male				AL, mm	SER, D^a^
DIMS (study 1)							
Overall (n = 115)	58	57	10.3 (1.5)	13 (12 to 14)	66.8 (14.2)	25.06 (24.92 to 25.20)	−4.02 (−4.23 to −3.81)
Less (n = 57)	30	27	10.2 (1.4)	13 (12 to 13)	55.9 (3.3)	25.09 (24.89 to 25.29)	−4.24 (−4.55 to −3.94)
More (n = 58)	28	30	10.3 (1.5)	13 (12 to 14)	77.6 (12.6)	25.01 (24.82 to 25.22)	−3.71 (−3.98 to −3.43)
SVL (study 2)							
Overall (n = 56)	29	27	10.8 (1.5)	14 (12 to 17)	63.3 (11.1)	24.84 (24.66 to 25.02)	−2.99 (−3.25 to −2.73)
Less (n = 28)	15	13	10.9 (1.7)	13.5 (13 to 14)	53.3 (6.1)	24.78 (24.54 to 25.01)	−2.96 (−3.34 to −2.59)
More (n = 28)	14	14	10.7 (1.4)	15.5 (12 to 17)	72.9 (4.0)	24.90 (24.62 to 25.18)	−3.02 (−3.38 to −2.65)

Abbreviations: AL, axial length; DIMS, defocus incorporated multiple segment; SER, spherical equivalent refraction; SVL, single vision lens.

^a^ Indicates significant main association of treatment type by analysis of covariance.

^b^ Percentage of time lockdown indicates the proportion of COVID-19 lockdown period between visits after in February 2020.

Both studies measured cycloplegic SER with an open-field autorefractor (NVision-K5001, Shin Nippon) and AL with a noncontact optical biometer (IOLMaster 500, Carl Zeiss Meditec AG). Five repeated readings were obtained. In the more clinically oriented study 1 (DIMS), cycloplegia was induced with 2 drops of 1% tropicamide, with administration of each drop separated by a 5-minute interval. In study 2 (SVL), 1 drop of 0.5% proparacaine HCL followed by 1 to 2 drops of 1% cyclopentolate HCL were administered for induction of cycloplegia. Although results from right and left eyes did not differ from each other, only data from the right eyes were used for data analysis.

### Statistical Analysis

The sample size had sufficient statistical power for detecting main associations of treatment type (0.97 and 0.93 for AL and SER, respectively) and lockdown severity (0.90 and 0.81 for AL and SER, respectively) over a 12-month study period. Two-way analysis of covariance was used to evaluate the treatment types (SVL vs DIMS) and the lockdown outcomes (less time vs more time) with the assumption of homogeneity of regression slopes of covariates between groups (eTable in the Supplement). Age and SER at baseline were used as covariates when comparing normalized change in AL and SER between treatment groups and lockdown severity. Statistical analyses were performed using SPSS statistical software version 22.0 (IBM), with significance set at *P* < .05. Data analysis was performed from June to July 2021.

## Results

In total, there were 115 participants (58 girls [50.4%]) in the DIMS group and 56 participants (29 girls [51.8%]) in the SVL group with valid baseline and follow-up data who were included in the current exploratory analysis. Table 1 lists the age, sex, AL, and SER at baseline. Differences were observed at baseline between the 2 studies for age and SER, with participants in the SVL group approximately half a year older (mean [SD] age, 10.3 [1.5] years vs 10.8 [1.5] years) and with milder myopia (mean [SD] baseline refraction, −2.99 [1.06] D vs −4.02 [1.46] D) than those in the DIMS group. The older age at baseline in the SVL group is likely due to rebaselining participants after their first study visit. In both studies, no significant baseline differences were observed between the participants included in the less lockdown time and more lockdown time cohorts.

In the total study populations, the unadjusted, normalized, 12-month mean (SD) change in AL was 0.21 (0.18) mm for the DIMS group and 0.26 (0.15) mm for the SVL group. The unadjusted, 12-month, mean (SD) normalized change in SER was −0.34 (0.49) D for the DIMS group and −0.50 (0.35) D for the SVL group.

**Figure 2.**
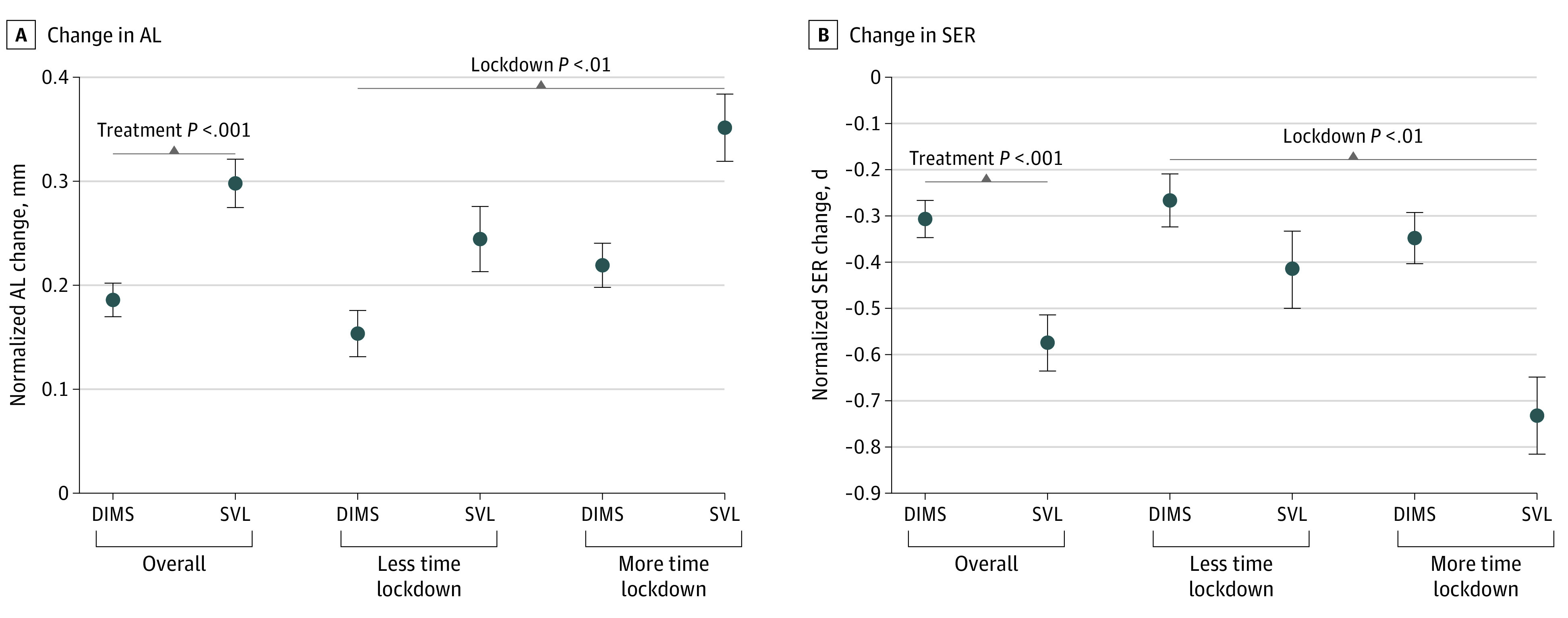
Covariate-Adjusted Mean 12-Month Normalized Change of Axial Length (AL) and Spherical Equivalent Refraction (SER) by Lockdown Severity in Children Wearing Defocus Incorporated Multiple Segments (DIMS) Lens (Study 1) and Single Vision Lens (SVL) (Study 2) Error bars represent standard errors of the mean.

The 2-way analysis of covariance analysis of AL change from baseline to follow-up adjusted for age and baseline refraction was overall significant (Figure 2). Both treatment type (covariate-adjusted estimate for DIMS vs SVL, 0.19 mm [95% CI, 0.16 to 0.22 mm] vs 0.30 mm [95% CI, 0.25 to 0.35 mm]; *P* < .001) and lockdown severity (less time vs more time, 0.20 mm [95% CI, 0.16 to 0.24 mm] vs 0.29 mm [95% CI, 0.25 to 0.32 mm]; *P* = .001) were significantly associated with changes in AL, without a significant interaction association (Figure 2). For the change in SER, the 2-way analysis of covariance was also overall significant, with significant associations with treatment type (DIMS vs SVL, −0.31 D [95% CI, −0.39 to −0.23 D] vs −0.57 D [95% CI, −0.69 to −0.45 D]; *P* = .001) and lockdown severity (less time vs more time, −0.34 D [95% CI, −0.44 to −0.25 D] vs −0.54 D [95% CI, −0.64 to 0.44 D]; *P* = .01). Again, no significant interaction association was observed (Figure 2). Hence, in this interstudy comparison, DIMS lens treatment was significantly associated with a slower 12-month progression of AL and SER of 34% and 46%, respectively, in the overall population, independent of the severity of the lockdown. Furthermore, in the more time lockdown subpopulation, DIMS lens treatment (AL, 0.22 mm [95% CI, 0.18 to 0.26 mm]; SER, −0.35 D [95% CI, −0.46 to −0.24 D]) was significantly associated with a slower 12-month progression of AL and SER of 37% and 52%, respectively, when compared with SVL (AL, 0.35 mm [95% CI, 0.29 to 0.42 mm]; SER, −0.73 D [95% CI, −0.89 to −0.57 D]). Table 2 shows the changes in SER and AL in studies^21,27^ that were conducted before COVID-19 in participants of similar baseline age and refraction.

**Table 2.** Historical Control (SVL) Data on Annual Progression Rates From the Prelockdown Period

Study	Years of study	Participants, No.	Baseline age, mean (SD), y	Follow-up period, mo	Mean (SD)	
					AL progression, mm	SER progression, D
Lam et al,^21^ 2014	2007-2009	63	10.9 (1.7)	24	0.37 (0.24)	−0.79 (0.56)
Lam et al,^27^ 2020	2014-2017	81	10.0 (1.5)	24	0.55 (0.18)	−0.85 (0.72)
Current SVL study	2019-2020	56	10.8 (1.5) (rebaselining)	12 (normalized)	0.29 (0.18)	−0.56 (0.46)

Abbreviations: AL, axial length; SER, spherical equivalent refraction; SVL, single vision lens.

## Discussion

To our knowledge, this cohort study is the first to evaluate the association between a myopic defocus treatment and myopia progression in longitudinal studies, intended to slow down myopia progression, that were conducted during the COVID-19 pandemic. According to the results, the COVID-19 lockdown time was associated with faster myopia progression and axial elongation among schoolchildren. In this analysis, the DIMS spectacle lens was associated with a slower myopia progression during the pandemic, resulting in 34% and 46% slower 12-month normalized AL and SER progression, respectively.

In February 2020, the Hong Kong government announced suspension of in-person school classes for all the primary and secondary schools in the city.^30^ To maintain teaching and learning activities, most of the schools implemented study-from-home policies and online courses, as the major teaching component, leading to an increase in screen time.^3,4,5^ The COVID-19 lockdown also discouraged and prohibited children from going outdoors, further limiting lifestyle options that have been shown to be effective in controlling myopia.^7,8,11^ The results of the current analysis, along with others,^14,15^ provide data sets from longitudinal cohort studies demonstrating that the lockdown policy resulted in increased myopia progression in school children. Of note, this analysis also suggests that despite the increased myopia progression during the COVID-19 lockdown, DIMS lenses were associated with a significant reduction in both SER and AL progression compared with SVL treatment.

A recent cross-sectional epidemiological study^28^ also suggested that the prevalence of myopia increased during the COVID-19 pandemic, and noncycloplegic photorefraction shifted to more myopic values compared with the years preceding the COVID-19 pandemic. Interestingly, the cross-sectional data suggested that pandemic-related effects were more severe in children younger than 9 years, consistent with the findings of the Sydney Adolescent Vascular and Eye study,^7^ which reported that time spent on near distance work at baseline was associated with myopia incidence over the 5-year follow-up in the younger cohort (aged 6 years at baseline) but not in the older cohort (aged 12 years at baseline). Conversely, Lam et al^27^ reported that DIMS was more effective in protecting older children from myopia progression. Importantly, if DIMS treatment is more effective in older children, its association with reduced myopia progression in the current analysis may be underestimated because the mean age of the DIMS group was younger than that of the SVL group. This may explain why the difference between DIMS and SVL groups in the current analysis is lower than in the previous study.^27^ It is also worth noting that previous longitudinal studies^14,15^ on refractive error change during the COVID-19 lockdown were targeting whole cohorts (ie, including children with and without myopia). From the current results on children with myopia, we speculate that the drastic change in environmental risk factors during the lockdown period^4^ were associated with myopic shift in people without myopia and myopia progression in those with myopia.

To the best of our knowledge, the current analysis is the first report to support that optical treatment using myopic defocus was significantly associated with slower myopia progression during the conditions of the COVID-19 pandemic, which were unfavorable to myopia progression (Table 2). Although low-dose atropine for myopia control was reported to have reduced effectiveness in a recent case series,^31^ additional research is required to understand whether other treatments for reducing myopia progression, including orthokeratology and specific soft contact lenses, also retained their efficacy during the pandemic. The results of the current analysis are expected to aid clinicians in managing schoolchildren with myopia who have experienced a drastic decrease in time spent outdoor and their mode of learning during the COVID-19 pandemic.

### Limitations

The current analysis is subject to the limitations of interstudy comparisons and exploratory, prespecified analyses. However, both studies were conducted in parallel at the same institution and both study populations were from the same geographical region (the city of Hong Kong), such that the potential for differences or biases resulting from geographical, temporal, or optometric practice variation between both studies is low. Nevertheless, careful interpretation of the current analysis is warranted because of the difference in sample size in each treatment type (studies 1 and 2), difference in baseline characteristics between both studies (Table 1), and different methods for cycloplegia, despite the cycloplegic effect being comparable between tropicamide and cyclopentolate in the population with myopia.^32^ Participants in the DIMS group were younger and more myopic at baseline, compared with those in the SVL group. Also, because the participants in the DIMS group were from families in need, it is likely that these families had, on average, lower socioeconomic status, which is traditionally considered as a risk factor for myopia.^10,12^ In addition, the outcomes associated with lockdown severity were observed similarly in both groups, strongly suggesting that baseline population differences did not affect this outcome. Although neither study was initially designed or powered for the exploratory, prespecified analysis presented here, the sample size had sufficient statistical power for detecting main associations of treatment type (0.97 and 0.93 for AL and SER, respectively) and lockdown severity (0.90 and 0.81 for AL and SER, respectively) over a 12-month study period, despite the unequal sample size of the 2 groups in this analysis.

## Conclusions

To our knowledge, this cohort study is the first analysis evaluating optical myopia control measures using longitudinal data collected during the COVID-19 pandemic. During the COVID-19 lockdown, myopia progression was significantly faster with the increased exposure to myopiagenic factors, compared with prepandemic conditions. In addition, optical treatment with DIMS lenses was significantly associated with slower myopia progression, despite the negative association between COVID-19 lockdown and myopia progression.

## Notes

Supplement. **eTable.** Homogeneity of Regression Slopes of Covariates in Treatment Type and Lockdown Effect
