# Supplementary material for: Evaluation of an Optical Defocus Treatment for Myopia Progression Among Schoolchildren During the COVID-19 Pandemic
Source: JAMA Netw Open. 2022 Jan 14;5(1):e2143781. doi: 10.1001/jamanetworkopen.2021.43781 (PMC8760616; doi:10.1001/jamanetworkopen.2021.43781)
Supplement: Supplement. — eTable. Homogeneity of Regression Slopes of Covariates in Treatment Type and Lockdown Effect [file jamanetwopen-e2143781-s001.pdf]

Supplemental Online Content

Choi KY, Chun RKM, Tang WC, To CH, Lam CSY, Chan HHL. Evaluation of an optical defocus treatment for myopia progression among schoolchildren during the COVID-19 pandemic. *JAMA Netw Open*. 2022;5(1):e2143781. doi:10.1001/jamanetworkopen.2021.43781

eTable. Homogeneity of Regression Slopes of Covariates in Treatment Type and Lockdown Effect

This supplemental material has been provided by the authors to give readers additional information about their work.

© 2022 Choi KY et al. *JAMA Network Open.*

eTable. Homogeneity of Regression Slopes of Covariates in Treatment Type and Lockdown Effect

		Raw B	p	Interaction – t test	p
Change in SER					
Age	*Treatment type*				
	DIMS	0.09	0.001	0.42	0.67
	SVL	0.07	0.03		
	Lockdown effect				
	Less	0.09	< 0.01	-0.55	0.58
	More	0.07	0.02		
Baseline SER	*Treatment type*				
	DIMS	0.01	0.85	0.14	0.89
	SVL	-0.00	0.94		
	Lockdown effect				
	Less	0.01	0.80	-0.98	0.33
	More	-0.04	0.21		
Change in AL					
Age	*Treatment type*				
	DIMS	-0.03	0.02	-0.11	0.92
	SVL	-0.03	< 0.01		
	Lockdown effect				
	Less	-0.04	< 0.01	0.39	0.70
	More	-0.03	< 0.01		
Baseline SER	*Treatment type*				
	DIMS	-0.01	0.31	0.98	0.33
	SVL	-0.03	0.08		
	Lockdown effect				
	Less	-0.01	0.53	0.21	0.84
	More	-0.01	0.62		

© 2022 Choi KY et al. *JAMA Network Open.*
